# Cervical spine alignment and clinical outcomes after multilevel anterior cervical decompression and fusion with or without plate: A minimal 5-year follow-up of a CONSORT-compliant article

**DOI:** 10.1097/MD.0000000000026126

**Published:** 2021-07-30

**Authors:** Yan Liang, Shuai Xu, Guanjie Yu, Zhenqi Zhu, Haiying Liu

**Affiliations:** Department of Spinal Surgery, Peking University People's Hospital, Peking University, No. 11 Xizhimen South Street, Xicheng District, Beijing, PR China.

**Keywords:** anterior cage-with-plate, anterior cervical discectomy and fusion, cervical alignment, cervical spondylotic myelopathy, self-locked stand-alone cage

## Abstract

It was reported imperative on cervical sagittal alignment reconstruction after anterior multilevel procedures with self-locked stand-alone cage (SSC) or anterior cage-with-plate (ACP) system multilevel while there was little knowledge about the relationship on cervical alignment and clinical outcomes.

To identify the importance of cervical sagittal alignment after 3-level anterior cervical discectomy and fusion on cervical spondylotic myelopathy with SSC and ACP system.

Seventy-seven patients with SSC system (SSC group) and 52 cases with ACP system (ACP group) from February 2007 to September 2013 were enrolled with well-matched demographics. Cervical alignment included C2–7 lordosis (CL), operated-segment cervical lordosis (OPCL), upper and lower adjacent-segment cervical lordosis, range of motion of upper and lower adjacent segment at preoperation, immediate postoperation, and the final follow-up. Clinical outcomes contained the neck disability index (NDI), the Japanese Orthopaedic Association (JOA) score, visual analogous scale (VAS) of arm and neck and adjacent segment degeneration (ASD). Patients were then divided into CL improved subgroup (IM subgroup) and non-improved subgroup (NIM subgroup).

There were improvements on CL and OPCL in both groups. The change of CL and OPCL larger in ACP group (*P* < .05) but upper adjacent-segment cervical lordosis/lower adjacent-segment cervical lordosis and range of motion of upper adjacent segment/range of motion of lower adjacent segment were of no significance. NDI, JOA, and VAS got improvement in both groups at immediate postoperation and the final follow-up while ASD was in no difference between SSC and ACP group. A total of 80 patients (39 vs 41) acquired CL improvement with a larger population in ACP group. There were no differences on the rate of ASD, NDI, JOA, VAS, and their change between IM and NIM subgroup. The changes of CL were not correlated to NDI, JOA, VAS, and their change.

SSC and ACP group both provide improved OPCL and efficacy on 3-level cervical spondylotic myelopathy with little impact on adjacent segment. The change of CL is not correlated to clinical outcomes.

## 1 Introduction

It was reported that anterior cervical discectomy and fusion (ACDF) has been considered a world-widely accepted procedure for the treatment of cervical spondylotic myelopathy (CSM).^[1,2]^ The anterior cage-with-plate system (ACP) consisting of polyetheretherketone cage with titanium plates can support the stability for cervical spine but it is probably with the side effects such as screw displacement, esophageal perforation, and dysphagia.^[3]^ Besides, some publication holds that anterior plating may also be associated with potential disadvantages and complications. In recent decades, the self-locked stand-alone cage (SSC), with 2 integrated self-locking clips and inserting into the vertebral body though the endplate, has been gradually applied for CSM.^[4,5]^ The comparisons on radiological and clinical outcomes between SSC and ACP system have been studied for years.^[4,6]^ While there was still a vague on efficacy for cervical alignment reconstruction between the 2 procedures, particularly in multilevel surgeries.

Cervical sagittal alignment was verified important parameters and reported probably correlated to regional disability and quality of life.^[7]^ In previous studies, one of the objectives for ACDF was to improve or rebuilt cervical alignment as it might be related to clinical outcomes.^[6–8]^ A variety of disorders of cervical spine might begin with alignment pathology and lead to aggressive abnormalities of the cervical spine,^[9]^ but others held suspicious attitude that the cervical sequence may be not associated with neurofunction and life quality.^[10,11]^ What's more, with the wider surgical filed, more iatrogenic interference and internal instrument in multilevel ACDF, there was little knowledge about the change of cervical alignment on the index segment and the whole spine, as well as the relationship between cervical alignment and clinical outcomes.

Consequently, it rises two points of controversies: is it comparable on cervical alignment and clinical outcomes for multilevel ACDF between multilevel SSC and ACP system? Does the reconstructed sagittal alignment lead to a long-term better quality of life after ACDF? Therefore, based on the patients with CSM underwent consecutive 3-level ACDF with SSC or ACP system, this study was to performe a minimum of 5-year follow-up.

## 2 Methods

### 2.1 Patient enrollment

It was a single-center retrospective study. The patients with CSM were selected based on the timing of presentation and then were divided into 2 groups from February 2007 to September 2013, where the patients performed with SSC was determined into SSC group while the cases with ACP system were defined as ACP group.

The inclusion criteria were patients with CSM required surgery with uncontrolled symptoms after at least 6-month conservation treatment; consecutive 3-level ACDF was performed; the surgery method was either in SSC group or ACP group; and intact radiologocal parameters could be obtained on X-ray and follow-up on clinical outcomes could be completed from all included patients. The exclusion criteria were followed by patients’ radiological parameters were too unclear to measure; patients with a history of previous cervical surgery; patients combined with other types of surgery such as artificial disc replacement or hybrid with SSC and ACP; patients underwent operation for cervical spine tumor, fracture, or infection; patients who underwent follow-up <5 years or unwilling to complete follow-up. All patients have signed informed consent. This study was approved by the Institutional Ethics Committee of our hospital (Approval No. 2018PHC076).

According to studies on similar parameters after SSC or ACP,^[3,6,7]^ the effect size |*ρ*| of all parameters ranged from 0.32 to 0.60 among patients with SSC. We defined the *α* error possibility was 0.05 and the power (1 − *β* error possibility) was 0.80, together with the estimation of loss rate of follow-rate was 20% to 30%, so the minimal sample of SSC group was 95. Then the sample of ACP group was determined with 1:1 to 1:2 matching to SSC group with propensity score matching methods. Therefore, a total of 180 participants (100 patients underwent SSC and 80 cases with ACP) were screened in the protocol. After a minimum 5-year follow-up, 77 patients were enrolled in SSC group and 52 patients were in ACP group with a follow-up rate of 71.7%. There were no significance in age, sex, and body mass index (BMI) between the 2 groups. The mean follow-up was 67.5 ± 5.2(m) (62–75 m) in SSC group and 69.2 ± 6.6(m) (60–77 m) in ACP group. As the index segment, C4–C7 occupied 77.9% in SSC group and 69.2% in ACP group (*P* = .267). There was no difference on blood loss but operation time was shorter in SSC group (*P* < .05) (Table 1).

**Table 1:** Information of demographics and ACDF on SSC group and ACP group.

Statistics	SSC group	ACP group	*P*
Sex (M: F)	41:36	27:25	.883
Age, yr	62.9 ± 8.8	63.5 ± 7.7	.846
BMI, kg/m^2^	24.1 ± 3.5	25.7 ± 3.1	.196
DM (n)	6	3	.658
Smoking (n)	13	8	.821
Follow-up (m)	67.5 ± 5.2 (62–75)	69.2 ± 6.6 (60–77)	.441
Operated level			.267
C3–C6 (n)	17	16	
C4–C7 (n)	60	36	
Operation duration, min	91.3 ± 16.6	115.4 ± 16.1	<.001
Blood loss, mL	67.4 ± 39.6	62.3 ± 32.7	.867

### 2.2 Surgical procedure

**Figure 1:**
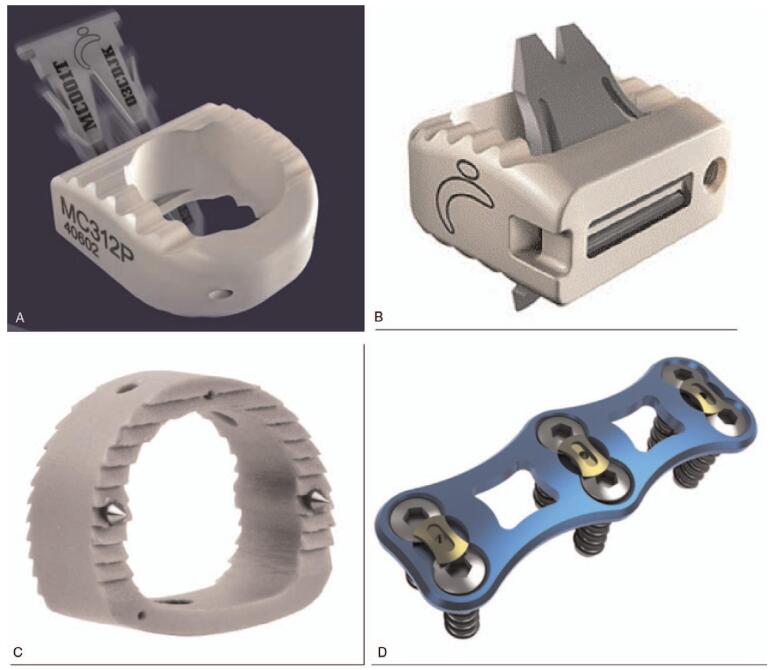
Different types of implants in SSC and ACP system. (A): zero-profile anchored spacer MC+; (B): zero-profile anchored spacer ROI-C; (C): solis cage; (D): anterior cervical plate. ACP = anterior cage-with-plate system, SSC = self-locked stand-alone cage.

All ACDF procedures were performed by the same senior surgeon. Patients were placed in a supine position after general anesthesia. A right-sided approach was utilized for both SSC and ACP group and standard Smith–Robinson approach^[12]^ to the cervical spine was performed. Bilateral discectomy and uncinated process resection was performed even with unilateral symptoms to remove osteophyte regrowth. After decompression completed, consecutive 3 cages were implanted orderly. In SSC group, the cages included zero profile anchored spacer MC+ (LDR, Troyes, France) and ROI-C (LDR, Troyes, France) while MC+ or Solis polyetheretherketone cages (Stryker, MI) combined with anterior plates (DePuySynthes, NJ) were applied for ACP group (Fig. 1). All patients were instructed to wear a soft collar for 2 months after ACDF.

### 2.3 Cervical alignment evaluation

**Figure 2:**
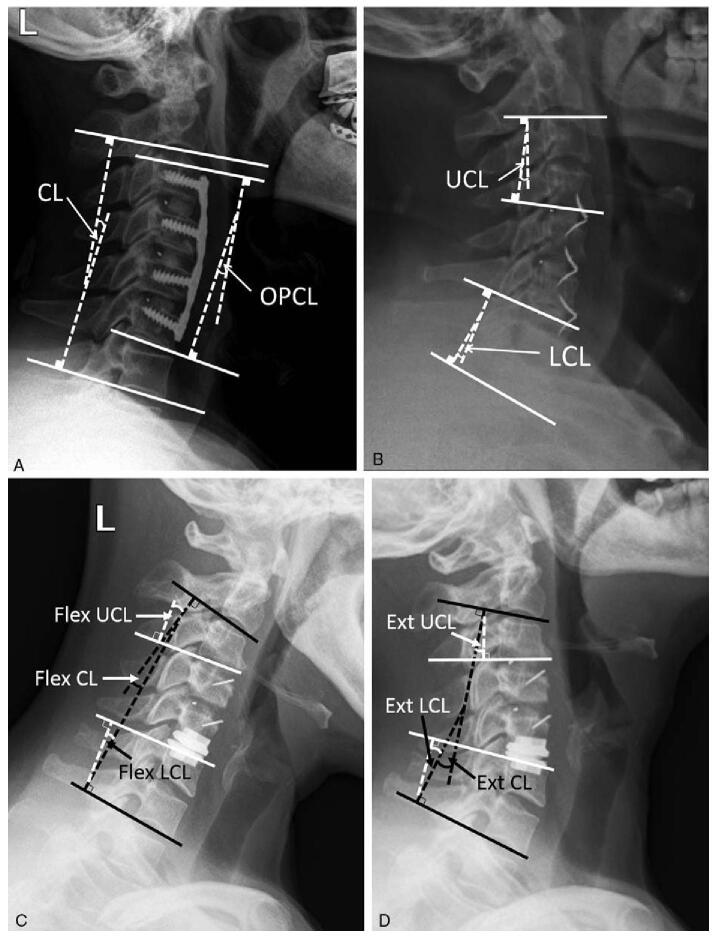
Measurements of cervical alignment on neutral lateral X-ray. (A): The measurements of CL and OPCL; (B): the measurements of UCL and LCL; (C, D): The measurements of UROM and LROM. CL = C2–7 lordosis, LCL = lower adjacent-segment cervical lordosis, LROM = lower adjacent-segment range of motion, OPCL = operated-segment cervical lordosis, UCL = upper adjacent-segment cervical lordosis, UROM = upper adjacent-segment range of motion.

Cervical alignment was mainly evaluated by C2–C7 lordosis (CL), as well as other measurements including operated-segment cervical lordosis (OPCL), upper and lower adjacent-segment cervical lordosis (UCL and LCL), the range of motion of upper and lower adjacent segment (UROM and LROM). CL was the angle from lower endplate of C2 to lower endplate of C7, the positive value was defined as cervical lordosis whereas the opposition means kyphosis; OPCL was the angle from the upper endplate of cranial operated vertebrae to lower endplate of distal operated vertebrae; UCL was the angle from upper endplate of the cranial vertebrae of upper adjacent segment to lower endplate of the distal vertebrae of upper adjacent segment, similar to LCL. UROM and LROM was the flextion-extention angle from upper endplate of the cranial vertebrae to lower endplate of the distal vertebrae of adjacent segment (Fig. 2). All parameters were measured from standard lateral plain radiographs, where CL, OPCL, UCL, and LCL were obtained at preoperation (POP), immediate postoperation (IPO) and the final follow-up (FFU), and UROM and LROM were measured at POP and FFU. All measurements were repeatedly measured by 3 dependent observers at each period.

### 2.4 Clinical outcomes assessment

The clinical outcomes were evaluated with the neck disability index (NDI) score, the Japanese Orthopaedic Association (JOA) score, and visual analogous scale (VAS) of arm and neck, which were both evaluated at POP, IPO, and FFU >5 years. The recovery rate (RR) of JOA was calculated by Hirabayashi method: RR (%) = (IPO or FFU JOA – POP JOA)/(17 – POP JOA) × 100. NDI was the indicator from 0 to 50 and a higher NDI meant a worse quality of life; a larger JOA score was corresponded to a better neurological function with a full mark of 17; VAS was from 0 to10, and the higher score represented more pain. In addition, complications such as reoperation and adjacent segment degeneration (ASD) were also recorded at IPO and FFU.

### 2.5 Subgroup analysis

We assumed that there were comparable outcomes between SSC and ACP groups with no much heterogeneity. After the data synthesis from SSC and ACP group, subgroup analysis was then performed based on the change of CL, which was calculated by CL at FFU minus CL at POP. An increase of CL represented improvement of cervical alignment while decrease of CL indicated deterioration of cervical spine sequence. At FFU, patients were divides into CL improved subgroup (IM subgroup) and non-improved subgroup (NIM subgroup).

### 2.6 Statistical analysis

All measurement data were expressed by mean ± standard deviation. The independent sample *t* test was used to compare cervical alignment parameters and clinical outcomes between SSC and ACP groups and between IM and NIM subgroups. Paired *t* test and variance analysis were used to compare outcomes among POP, IPO, and FFU within the same group. Chi-squared test or Fisher test was performed on dichotomous between the 2 groups. Pearson correlation analysis was utilized for the change of CL and clinical outcomes at FFU, respectively. Intraobserver reproducibility of these measurements was explored with the intraclass correlation coefficient (ICC). On interobserver reliability, the ICC with 95% CI was also identified, comparing the mean of all 3 measurements from 3 observers. ICC < |0.40| indicated poor results; |0.40| to |0.75| was fair to good, and |0.75| to |1.00| was excellent reliability. The statistical analysis was performed using SPSS 22.0 (International Business Machines Corporation, Armonk, NY) and statistical significance was defined as *P* < .05.

## 3 Results

Intraobserver reproducibility and interobserver reliability using ICC for all radiological parameters showed good to excellent agreement (Table 2).

**Table 2:** Intra-observer reproducibility and inter-observer reliability using ICC for all parameters.

	POP		IPO		FFU	
Parameters	Intra-observer	Inter-observer	Intra-observer	Inter-observer	Intra-observer	Inter-observer
CL	0.87	0.79	0.83	0.76	0.93	0.88
OPCL	0.92	0.77	0.89	0.81	0.91	0.89
UCL	0.92	0.71	0.72	0.70	0.88	0.71
LCL	0.88	0.81	0.78	0.78	0.89	0.79
UROM	0.79	0.73	0.81	0.71	0.76	0.75
LROM	0.86	0.76	0.79	0.76	0.81	0.75

### 3.1 Comparisons between SSC and ACP groups

There were no significant differences on CL between SSC and ACP group at POP, IPO and FFU (all *P* > .05), but the change of CL at FFU was larger in ACP group (*P* = .026). The CL was improved in ACP group at FFU contrasted with POP but not at IPO. OPCL were of no differences between the 2 groups. In addition, there were improvements on OPCL at IPO in both groups and at FFU in ACP group (*P* = .047, *P* = .007, and *P* = .019, respectively). There was a significance on UCL at IPO between 2 groups (*P* = .037) but not at POP and FFU. There were no statistical differences on LCL between the 2 groups and at each period except for a less LCL in ACP group at FFU (*P* = .044). There were no statistical differences on UROM and LROM between SSC and ACP group all the time. While both UROM and LROM increased at FFU compared with POP in the 2 groups (Table 3).

**Table 3:** Comparisons on CL, OPCL, UCL, and LCL between SSC and ACP groups.

Parameters	SSC group	ACP group	*P*
CL at POP, °	9.1 ± 12.5	3.3 ± 11.7	.166
CL at IPO, °	12.8 ± 10.5	8.6 ± 9.8	.259
CL at FFU, °	13.1 ± 8.2	11.9 ± 10.5^‡^	.728
ΔCL, °^§^	4.0 ± 5.8	8.6 ± 7.9	.026
OPCL at POP, °	3.6 ± 9.1	2.9 ± 10.9	.747
OPCL at IPO, °	9.6 ± 9.0^∗^	11.9 ± 6.0^†^	.414
OPCL at FFU, °	7.9 ± 7.7	11.3 ± 5.9^‡^	.263
ΔOPCL, °^§^	4.1 ± 6.0	10.2 ± 8.8	.014
UCL at POP, °	4.6 ± 7.2	3.4 ± 6.7	.645
UCL at IPO, °	2.8 ± 4.8	-0.8 ± 3.5	.037
UCL at FFU, °	4.8 ± 6.2	1.1 ± 5.1	.120
ΔUCL, °^§^	-0.5 ± 5.8	-2.0 ± 5.7	.471
LCL at POP, °	4.6 ± 5.9	1.5 ± 6.1	.178
LCL at IPO, °	5.2 ± 6.1	0.5 ± 10.7	.145
LCL at FFU, °	7.2 ± 6.7	0.7 ± 9.4	.044
ΔLCL, °^§^	2.4 ± 5.5	-1.1 ± 5.3	.083
UROM at POP, °	9.3 ± 3.5	10.5 ± 6.2	.586
UROM at FFU, °	12.8 ± 3.8^†^	13.6 ± 4.7^†^	.616
ΔUROM, °^§^	3.7 ± 4.6	3.3 ± 7.7	.864
LROM at POP, °	5.8 ± 4.5	6.4 ± 3.6	.721
LROM at FFU, °	10.1 ± 3.2^‡^	9.9 ± 5.6^†^	.344
ΔLROM, °^§^	4.8 ± 6.7	3.1 ± 5.5	.297

There were no differences in NDI between SSC and ACP group at POP, IPO, and FFU, so were their change. While there were statistical differences at IPO compared with POP, and a further improvement at FFU compared with IPO (all *P* < .01). JOA and the RR of JOA were of no differences between SSC and ACP group, but there was also an improvement at IPO and FFU compared with POP (all *P* < .01). Similarly, VAS of arm and neck both got improvement at IPO and further improvement at FFU, whichever the group (all *P* < .01) (Table 4).

**Table 4:** Comparisons on clinical outcomes between SSC and ACP groups.

Parameters	SSC group	ACP group	*P*
NDI at POP	37.6 ± 2.8	38.5 ± 3.2	.406
NDI at IPO	19.9 ± 8.6^∗^	18.4 ± 4.1^∗^	.579
NDI at FFU	12.5 ± 9.9^†^^,^^‡^^,^^§^	12.5 ± 5.9^†^^,^^‡^^,^^§^	.992
Δ_1_NDI^||^	17.9 ± 8.5	20.4 ± 4.1	.364
Δ_2_NDI^||^	25.3 ± 10.4	26.3 ± 4.5	.759
JOA at POP	10.5 ± 1.7	10.1 ± 2.1	.561
JOA at IPO	14.5 ± 1.7^∗^	14.8 ± 0.9^∗^	.616
JOA at FFU	15.9 ± 2.2^†^	15.5 ± 1.6^†^^,^^‡^	.565
RR_1_ of JOA (%)^||^	60.3 ± 30.0	68.2 ± 11.1	.399
RR_2_ of JOA (%)^||^	82.7 ± 36.3	80.6 ± 19.0	.865
VAS of arm at POP	8.1 ± 0.9	7.8 ± 1.2	.447
VAS of arm at IPO	3.5 ± 1.1^∗^	2.5 ± 0.8^∗^	.019
VAS of arm at FFU	0.7 ± 1.5^†^^,^^‡^^,^^§^	0.7 ± 1.7^†^^,^^‡^^,^^§^	.999
Δ_1_VAS of arm^||^	4.8 ± 1.3	5.3 ± 1.1	.333
Δ_2_VAS of arm^||^	7.6 ± 1.7	7.1 ± 1.9	.470
VAS of neck at POP	5.4 ± 1.6	6.0 ± 1.7	.340
VAS of neck at IPO	2.1 ± 1.6^∗^	2.3 ± 1.2^∗^	.719
VAS of neck at FFU	0.7 ± 2.1^†^^,^^‡^^,^^§^	0.8 ± 1.3^†^^,^^‡^^,^^§^	.808
Δ_1_VAS of neck^||^	3.4 ± 1.5	3.8 ± 1.4	.459
Δ_2_VAS of neck^||^	4.9 ± 2.3	5.3 ± 1.8	.564

There were no cases performed secondary operation in both groups except for 1 patient in SSC group with a second-stage surgery in posterior approach 2 weeks later after ACDF. The fusion rates were both 100% in the 2 groups at FFU. The fracture and slight displacement of 1 plate-screw occurred at IPO but it was stasis without any clinical symptom at FFU. There were respectively 3.9% (3/77) and 9.6% (5/52) cases with dysphagia in SSC and ACP group (*P* = .186) while no case occurred at FFU. There were 63.6% cases with ASD in SSC group at FFU while 61.5% in ACP group (*P* = .809), where ASD in upper adjacent segment occurred in 17 cases in SSC group and 10 in ACP group (*P* = .748) and ASD in lower adjacent segment occurred in 31 cases in SSC group and 18 in ACP group (*P* = .528).

### 3.2 Comparisons between IM and NIM subgroups

In total, there were 79 patients with cervical lordosis at baseline with no difference in SSC (49/77) and ACP (30/52) group (*P* = .497); after ACDF, 78 cases maintained lordosis in the 2 groups (48 vs 30) (*P* = .579) and 43 patients got alignment-correction in SSC (22/77) and ACP (21/52) group with no difference (*P* = .163). Eventually, a total of 80 patients (39 vs 41) acquired CL-increase with a larger population in ACP group (*P* = .001).

**Figure 3:**
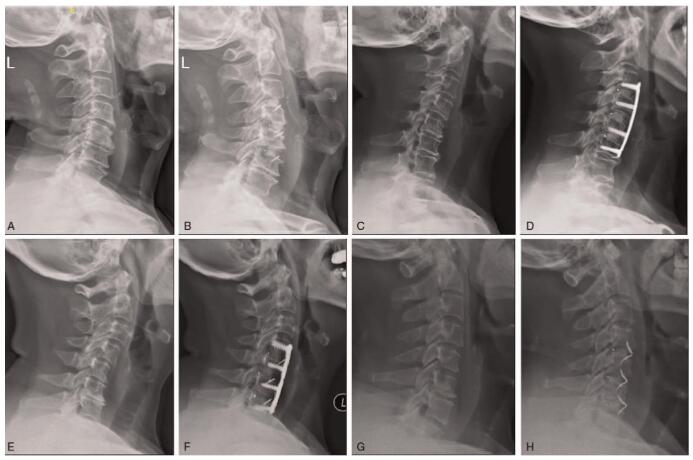
Typical cases referred to CL and clinical outcomes. (A, B): The X-ray at POP and FFU of a 76-year-old man with the largest positive CL. The case from SSC group performed C3–6 with MC+ showed CL was 29.7° with NDI of 9, ΔNDI of 27, JOA of 16, JOA-RR of 85.7%, arm-VAS of 0, and neck-VAS of 0 at FFU. (C, D): The X-ray at POP and FFU of a 64-year-old woman with the largest negative CL. The case from ACP group performed C3–6 with Solis cage+plate showed CL was –8.2° with NDI of 6, ΔNDI of 27, JOA of 17, JOA-RR of 100%, arm-VAS of 0 and neck-VAS of 0 at FFU; (E, F): the X-ray at POP and FFU of a 67-year-old woman with the largest improvement of CL. The case from ACP group and IM subgroup performed C4–7 with MC+ cage+plate showed ΔCL was 26° with NDI of 15, ΔNDI of 26, JOA of 15, JOA-RR of 75%, arm-VAS of 1 and neck-VAS of 2 at FFU; (G, H): the X-ray at POP and FFU of a 52-year-old man with the largest decrease of CL. The case from SSC group and NIM subgroup performed C4–7 with ROI-C showed ΔCL was –11.3° with NDI of 11, ΔNDI of 31, JOA of 17, JOA-RR of 100%, arm-VAS of 1, and neck-VAS of 0 at FFU. ACP = anterior cage-with-plate system, CL = C2–7 lordosis, FFU = the final follow-up, IM = improvement, JOA = the Japanese Orthopaedic Association, NDI = the neck disability index, NIM = no improvement, POP = preoperation, RR = recovery rate, SSC = self-locked stand-alone cage. Δ means the change of parameters at FFU compared to POP.

There were 81 cases in IM subgroup while 48 cases in NIM subgroup. Table 5 showed there were no differences on NDI, the change of NDI, JOA, RR of JOA, VAS of arm and neck as well as their change at FFU between the 2 subgroups, so was ASD (all *P* > .05). The largest increase of CL was 26° in the case from ACP group with a NDI improvement of 26, JOA RR of 75%, arm-VAS improvement of 7 and neck-VAS improvement of 5, while the case with the largest decrease of –11.3° in NIM subgroup also showed a NDI improvement of 31, JOA RR of 100%, arm-VAS improvement of 9, and neck-VAS improvement of 5 (Fig. 3). According to correlation analysis, there was no correlation between the change of CL and NDI, JOA, VAS of arm, VAS of neck as well as their change (*P* > .05) (Table 6).

**Table 5:** Comparisons on clinical outcomes between IM and NIM subgroups.

Parameters	IM subgroup	NIM subgroup	*P*
NDI at FFU	11.9 ± 6.8	13.7 ± 11.0	.618
ΔNDI	26.4 ± 6.7	24.6 ± 11.0	.592
JOA at FFU	15.9 ± 1.5	15.4 ± 2.7	.575
RR of JOA (%)	84.1 ± 20.6	77.2 ± 43.2	.578
VAS of arm at FFU	0.56 ± 1.54	0.89 ± 1.69	.612
ΔVAS of arm	7.33 ± 1.81	7.44 ± 1.88	.883
VAS of neck at FFU	1.00 ± 2.06	0.22 ± 0.44	.277
ΔVAS of neck	5.22 ± 2.34	4.78 ± 1.30	.603
ASD (n)	48	33	.281

**Table 6:** Pearson correlation analysis between clinical outcomes and the change of CL.

	*r*	*P*
Δ^∗^CL and NDI at FFU	–0.164	.412
ΔCL and ΔNDI	0.162	.420
ΔCL and JOA at FFU	0.213	.285
ΔC and RR of JOA	0.215	.282
Δ^∗^CL and VAS of arm at FFU	-0.296	.134
ΔCL and ΔVAS of arm	0.183	.361
Δ^∗^CL and VAS of neck at FFU	0.255	.200
ΔCL and ΔVAS of neck	0.115	.567

## 4 Discussion

ACDF allows direct decompression, reconstruction of cervical lordosis, and stabilization of the operated segments by anterior approach.^[13]^ The SSC system, as a neo-designed implant, has been widely applied for CSM, but most studies mainly concentrated on the comparison between short-level SSC and ACP in terms of complications and clinical outcomes by a short- to middle- term cohort.^[14,15]^ Yun et al^[16]^ conduct a 2-year follow-up on 2-level contiguous ACDF and showed comparable clinical outcomes and capacity of lordosis-maintenance between SSC and ACP. However, Shi et al^[17]^ described a favorable outcomes on SSC on complications for 3-level CSM with 3-year follow-up. With different design concept between SSC and ACP system, the mechanism on cervical alignment reconstruction and on operated- or adjacent-segment effect was still full of challenge. Therefore, this study focused on more elaborated measurements on global cervical alignment, operated- and adjacent- segment lordosis, as well as ASD. It firstly, with a long-term visit, demonstrated that ACP was slightly superior to SSC on CL and OPCL improvement, but both with little impact on adjacent segment.

Generally, restoration of CL was achieved by posterior osteophytectomy, opening of the posterior longitudinal ligament, and the size or shapes of implants.^[6,16]^ Our series addressed an optimistic outcome on CL improvement by SSC and ACP. When referring to restoration of CL between the 2 approaches, Chen et al^[4]^ indicated SSC was inferior and may not provide better sagittal CL reconstruction in 3-level fixation with 24 to 36 m follow-up. It was probably with the explanation that the titanium alloy plate was positioned in anterior vertebral line and the anterior intervertebral disc height might decrease less than posterior one. In addition, the CL was restored by pulling the involved vertebrae towards the prebent lordotic ventral plate, which could make the segmental angle more improved.^[6]^ While the zero-profile anchored spacer, consisting of a cage and single or two anchoring clips, showed less ability to restore CL contrasted with ACP.^[18]^

There was decrease on UCL and LCL in ACP group at IPO and FFU, which was considered a compensation by the adjacent segment for a larger OPCL with ACP system in case to keep appropriate cervical lordosis. Secondly, studies on biomechanics revealed that SSC provided less stiffness of cervical spine as locking plate does in 2 or 3-level instrumentation.^[19]^ As a consequence, SSC system was more closely matched to the physiological elastic modulus of the vertebrae and exerted greater load transfer to the interbody cages. The excessive stiffness of the metallic plate may incur stress shielding and uncomfortable kinematics on adjacent segments.^[20,21]^ In addition, accurate anterior osteophytectomy and plate-bed preparation was essential to smoothen anterior surface of the adjacent vertebrae for ACP, which has also been advocated as a possible risk factor on the loss of UCL and LCL.^[22]^ In total, however, there was no much influence on adjacent segment lordosis in both approaches in this study.

One concern on multilevel ACDF was the potential appearance of ASD with increased rigidity. Studies have shown that the presence of a plate was more likely to accelerate degenerative changes in adjacent segments.^[16,23]^ However, a meta-analysis performed by Zhang et al^[24]^ showed there was no statistical difference in the incidence of between the multilevel SSC and ACP groups with a short-term follow-up. Therefore, the specific cause of ASD was still filled with confounding. Through a long-term follow-up, our series provided comparable incidence of ASD between SSC and ACP with a rate of >60%. While it was reported multilevel procedures may not be at greater risk of developing ASD compared with single-level procedure.^[14]^ Besides, the fact that no case with adjacent segment pathology for secondary surgery in either group and no obvious change of UCL and LCL indicated the 2 approaches put little and comparable impact on the progression of ASD.

The comparisons on short-term clinical outcomes between SSC and ACP remained controversial. Njoku et al^[8]^ and Yan et al^[2]^ favored SSC with a better effect on pain relief and neurological function recovery. While Tong et al^[25]^ showed SSC and ACP exerted similar efficacy in improving the radiological outcomes and quality of life through a 2-year follow-up by a meta-analysis. Our data showed a statistical improvement in clinical outcomes at FFU contrasted with POP and IPO. The explanation might be that appropriate physiotherapy after surgery, subjective adaption of patients, and edema elimination of nerve root would promote a further step on qualified adjusted life year.

It was debatable that the improvement and preservation of CL was the key goal after multilevel ACDF. Sagittal malalignment after ACDF may cause postoperative axial pain and worsening of neurologic deficits.^[21]^ However, a meta-analysis performed by Luo et al^[11]^ showed there was no correlation between clinical outcomes and cervical sagittal alignment. Our study addressed that the patients could acquire long-term and satisfactory clinical function recovery whether with CL-improvement or not. Indeed, was it really important to get an improvement of CL? Posterior approaches such as laminoplasty were usually performed to achieve multilevel compression, but they were prone to be accompanied with postoperative complications and loss of cervical lordosis because of excessive posterior muscle stripping and impairment of muscle-ligament complex, which was associated with axial pain.^[26]^ While ACDF kept the superiority in minimal incision and invasion, interstitial-space approach and preservation of posterior complex, consequently leading to little kyphosis-derived symptom. Despite restoration of lordosis in a kyphotic cervical spine being a goal of surgery, there was no consensus about the optimal threshold of CL to achieve.^[27]^ Moreover, it was reported that the increased rigidity of adjacent segment and abnormal sagittal balance might promots ASD after ACDF.^[28]^ While in our study, neither in ACP group nor in NIM subgroup, the incidence of ASD was higher in contrast to another, which administrated ASD might not be directly derived from increased rigidity of adjacent segment or sagittal imbalance.

In this study, no identified correlation was drawn between clinical outcomes and the change of CL after both procedures. Despite with various types of cage and fixation, both procedures decompressed spinal cord directly by removing the anterior pathogenic compression,^[24]^ so almost all patients acquired benefit from whichever the group. Although the volume of spinal canal could be effected by CL,^[7]^ there was compensatory space for spinal cord recruitment after adequate decompression. Then, most patients suffered from severe dysfunction with long history before ACDF and they could acquire instantly complaint relief after surgery by compression removal instead of what the change of CL brought. Moreover, for most cases, the change of CL, although with CL increase or decrease, was in a acceptable range, which might be below the threshold of accelerating alignment-related dysfunction.^[8]^ Therefore, the improvement of CL after 3-level ACDF with SSC or ACP seemed not so essential as reported.

There were some limitations in our study. Firstly, the sample of both groups were little since only patients with CSM performed 3-level ACDF were enrolled. Then, global cervical alignment was expressed by CL, which could not ideally describe total shapes of cervical spine such as sigmoid-S-type since it might effect the spinal canal volume^[29]^ and result in a shape-derived symptom. Finally, the conclusion was suitable for 3-level ACDF on CSM but might not for other types such as cervical spondylotic radiculopathy, where the nerve root could be effected by the height of intervertebral foramen result from the change of cervical alignment.

## 5 Conclusion

Based on the patients with CSM performed 3-level ACDF, both SSC and ACP system provide a long-term and effective outcomes in maintaining and restoration of CL, OPCL, and quality of life, and the procedures make little impact on adjacent segment. ACP system was slightly superior to SSC in CL improvement. There is no identified correlation between clinical outcomes and the change of CL, so the improvement of CL after 3-level ACDF seemed not so essential as reported.

## Acknowledgments

The authors acknowledge Houshan Lv who contributed towards the study by making substantial contributions to the design and the acquisition of data.

## Author contributions

**Conceptualization:** Yan Liang, Haiying Liu.

**Data curation:** Shuai Xu, Guanjie Yu, Haiying Liu.

**Formal analysis:** Yan Liang, Shuai Xu.

**Investigation:** Yan Liang, Guanjie Yu.

**Methodology:** Yan Liang, Shuai Xu, Zhenqi Zhu.

**Project administration:** Haiying Liu.

**Resources:** Shuai Xu, Zhenqi Zhu.

**Software:** Yan Liang, Shuai Xu.

**Validation:** Yan Liang.

**Visualization:** Haiying Liu.

**Writing – original draft:** Yan Liang, Shuai Xu, Haiying Liu.

**Writing – review & editing:** Yan Liang, Shuai Xu, Haiying Liu.

## References

[1] Lau] D Chou D Mummaneni PV. Two-level corpectomy versus three-level discectomy for cervical spondylotic myelopathy: a comparison of perioperative, radiographic, and clinical outcomes. J Neurosurg Spine 2015;23:280–9. 26091438 10.3171/2014.12.SPINE14545

[2] Yan B Nie L. Clinical comparison of Zero-profile interbody fusion device and anterior cervical plate interbody fusion in treating cervical spondylosis. Int J Clin Exp Med 2015;8:13854–8. 26550337 PMC4613022

[3] Tasiou A Giannis T Brotis AG. Anterior cervical spine surgery-associated complications in a retrospective case-control study. J Spine Surg 2017;3:444–59. 29057356 10.21037/jss.2017.08.03 PMC5637201

[4] Chen Y Liu Y Chen H. Comparison of curvature between the zero-P spacer and traditional cage and plate after 3-level anterior cervical discectomy and fusion: mid-term results. Clin Spine Surg 2017;30:E1111–6. 27642818 10.1097/BSD.0000000000000440

[5] Scholz M Schnake KJ Pingel A. A new zero-profile implant for stand-alone anterior cervical interbody fusion. Clin Orthop Relat Res 2011;469:666–73. 20882376 10.1007/s11999-010-1597-9 PMC3032850

[6] Albanese V Certo F Visocchi M. Multilevel anterior cervical diskectomy and fusion with zero-profile devices: analysis of safety and feasibility, with focus on sagittal alignment and impact on clinical outcome: single-institution experience and review of literature. World Neurosurg 2017;106:724–35. 28625909 10.1016/j.wneu.2017.06.051

[7] Ames CP Blondel B Scheer JK. Cervical radiographical alignment: comprehensive assessment techniques and potential importance in cervical myelopathy. Spine (Phila Pa 1976) 2013;38:S149–60. 24113358 10.1097/BRS.0b013e3182a7f449

[8] Njoku IJ Alimi M Leng LZ. Anterior cervical discectomy and fusion with a zero-profile integrated plate and spacer device: a clinical and radiological study: clinical article. J Neurosurg Spine 2014;21:529–37. 25105338 10.3171/2014.6.SPINE12951

[9] Ames CP Smith JS Eastlack R. Reliability assessment of a novel cervical spine deformity classification system. J Neurosurg Spine 2015;23:673–83. 26273762 10.3171/2014.12.SPINE14780

[10] Sun Y Li L Zhao J. Comparison between anterior approaches and posterior approaches for the treatment of multilevel cervical spondylotic myelopathy: a meta-analysis. Clin Neurol Neurosurg 2015;134:28–36. 25935128 10.1016/j.clineuro.2015.04.011

[11] Luo J Cao K Huang S. Comparison of anterior approach versus posterior approach for the treatment of multilevel cervical spondylotic myelopathy. Eur Spine J 2015;24:1621–30. 25840781 10.1007/s00586-015-3911-4

[12] Smith GW Robinson RA. The treatment of certain cervical-spine disorders by anterior removal of the intervertebral disc and interbody fusion. J Bone Joint Surg Am 1958;40-A:607–24. 13539086

[13] Song KJ Taghavi CE Lee KB. The efficacy of plate construct augmentation versus cage alone in anterior cervical fusion. Spine (Phila Pa 1976) 2009;34:2886–92. 19949367 10.1097/BRS.0b013e3181b64f2c

[14] Basques BA Louie PK Mormol J. Multi- versus single-level anterior cervical discectomy and fusion: comparing sagittal alignment, early adjacent segment degeneration, and clinical outcomes. Eur Spine J 2018;27:2745–53. 29946938 10.1007/s00586-018-5677-y

[15] Yang L Gu Y Liang L. Stand-alone anchored spacer versus anterior plate for multilevel anterior cervical diskectomy and fusion. Orthopedics 2012;35:e1503–10. 23027488 10.3928/01477447-20120919-20

[16] Yun DJ Lee SJ Park SJ. Use of a zero-profile device for contiguous 2-level anterior cervical diskectomy and fusion: comparison with cage with plate construct. World Neurosurg 2017;97:189–98. 27671883 10.1016/j.wneu.2016.09.065

[17] Shi S Liu ZD Li XF. Comparison of plate-cage construct and stand-alone anchored spacer in the surgical treatment of three-level cervical spondylotic myelopathy: a preliminary clinical study. Spine J 2015;15:1973–80. 25912505 10.1016/j.spinee.2015.04.024

[18] Scholz M Schleicher P Pabst S. A zero-profile anchored spacer in multilevel cervical anterior interbody fusion: biomechanical comparison to established fixation techniques. Spine (Phila Pa 1976) 2015;40:E375–80. 25584947 10.1097/BRS.0000000000000768

[19] Clavenna AL Beutler WJ Gudipally M. The biomechanical stability of a novel spacer with integrated plate in contiguous two-level and three-level ACDF models: an in vitro cadaveric study. Spine J 2012;12:157–63. 22405617 10.1016/j.spinee.2012.01.011

[20] Ciccone WN Motz C Bentley C. Bioabsorbable implants in orthopaedics: new developments and clinical applications. J Am Acad Orthop Surg 2001;9:280–8. 11575907 10.5435/00124635-200109000-00001

[21] Liu Y Wang H Li X. Comparison of a zero-profile anchored spacer (ROI-C) and the polyetheretherketone (PEEK) cages with an anterior plate in anterior cervical discectomy and fusion for multilevel cervical spondylotic myelopathy. Eur Spine J 2016;25:1881–90. 26968876 10.1007/s00586-016-4500-x

[22] Pitzen TR Chrobok J Stulik J. Implant complications, fusion, loss of lordosis, and outcome after anterior cervical plating with dynamic or rigid plates: two-year results of a multi-centric, randomized, controlled study. Spine (Phila Pa 1976) 2009;34:641–6. 19287352 10.1097/BRS.0b013e318198ce10

[23] Park JB Cho YS Riew KD. Development of adjacent-level ossification in patients with an anterior cervical plate. J Bone Joint Surg Am 2005;87:558–63. 15741622 10.2106/JBJS.C.01555

[24] Zhang D Liu B Zhu J. Comparison of clinical and radiologic outcomes between self-locking stand-alone cage and cage with anterior plate for multilevel anterior cervical discectomy and fusion: a meta-analysis. World Neurosurg 2019;125:e117–31. 30677575 10.1016/j.wneu.2018.12.218

[25] Tong MJ Xiang GH He ZL. Zero-profile spacer versus cage-plate construct in anterior cervical diskectomy and fusion for multilevel cervical spondylotic myelopathy: systematic review and meta-analysis. World Neurosurg 2017;104:545–53. 28526640 10.1016/j.wneu.2017.05.045

[26] Wang B Lu G Kuang L. Anterior cervical discectomy and fusion with stand-alone anchored cages versus posterior laminectomy and fusion for four-level cervical spondylotic myelopathy: a retrospective study with 2-year follow-up. BMC Musculoskelet Disord 2018;19:216–22. 30001719 10.1186/s12891-018-2136-1 PMC6043970

[27] McAviney J Schulz D Bock R. Determining the relationship between cervical lordosis and neck complaints. J Manipulative Physiol Ther 2005;28:187–93. 15855907 10.1016/j.jmpt.2005.02.015

[28] Bydon M Xu R De la Garza-Ramos R. Adjacent segment disease after anterior cervical discectomy and fusion: Incidence and clinical outcomes of patients requiring anterior versus posterior repeat cervical fusion. Surg Neurol Int 2014;5:S74–8. 24843815 10.4103/2152-7806.130676 PMC4022999

[29] Sessumpun K Paholpak P Hindoyan KN. Characteristics of cervical spine motion in different types of cervical alignment: kinematic MRI study. Clin Spine Surg 2018;31:E239–44. 29315118 10.1097/BSD.0000000000000605

